# Case Report: Profound newborn leukopenia related to a novel RAC2 variant

**DOI:** 10.3389/fped.2024.1365187

**Published:** 2024-03-07

**Authors:** Geoffrey Hall, Ágnes Donkó, Cristina Pratt, Julie J. Kim-Chang, Paul L. Martin, Amy P. Stallings, John W. Sleasman, Steven M. Holland, Amy P. Hsu, Thomas L. Leto, Talal Mousallem

**Affiliations:** ^1^ Division of Allergy and Immunology, Department of Pediatrics, Duke University, Durham, NC, United States; ^2^ Molecular Defenses Section, Laboratory of Clinical Immunology and Microbiology (LCIM), National Institute of Allergy and Infectious Disease (NIAID), National Institutes of Health (NIH), Bethesda, MD, United States; ^3^ Division of Pediatric Transplant and Cellular Therapy, Department of Pediatrics, Duke University, Durham, NC, United States; ^4^ Immunopathogenesis Section, Laboratory of Clinical Immunology and Microbiology (LCIM), National Institute of Allergy and Infectious Diseases (NIAID), National Institutes of Health (NIH), Bethesda, MD, United States

**Keywords:** severe combined immunodeficiency, reticular dysgenesis, RAC2, inborn error of immunity, novel variant

## Abstract

We report the case of a 1-week-old male born full-term, who had two inconclusive severe combined immunodeficiency (SCID) newborn screens and developed scalp cellulitis and *Escherichia coli* bacteremia. He did not pass early confirmatory hearing screens. Initial blood counts and lymphocyte flow cytometry revealed profound neutropenia and lymphopenia with a T-/B-/NK- phenotype. Red blood cell adenosine deaminase 1 activity was within normal limits. A presumptive diagnosis of reticular dysgenesis was considered. Granulocyte colony-stimulating factor was started, but there was no improvement in neutrophil counts. Subsequent lymphocyte flow cytometry at around 4 weeks of age demonstrated an increase in T-, B- and NK-cell numbers, eliminating suspicion for SCID and raising concern for congenital neutropenia and bone marrow failure syndromes. Genetic testing revealed a novel variant in *RAC2* [c.181C>A (p.Gln61Lys)] (Q61K). RAC2, a Ras-related GTPase, is the dominant RAC protein expressed in hematopoietic cells and is involved with various downstream immune-mediated responses. Pathogenic *RAC2* variants show significant phenotypic heterogeneity (spanning from neutrophil defects to combined immunodeficiency) across dominant, constitutively activating, dominant activating, dominant negative, and autosomal recessive subtypes. Given the identification of a novel variant, functional testing was pursued to evaluate aberrant pathways described in other *RAC2* pathogenic variants. In comparison to wild-type RAC2, the Q61K variant supported elevated superoxide production under both basal and PMA-stimulated conditions, increased PAK1 binding, and enhanced plasma membrane ruffling, consistent with other dominant, constitutively active mutations. This case highlights the diagnostic challenge associated with genetic variants identified via next-generation sequencing panels and the importance of functional assays to confirm variant pathogenicity.

## Introduction

Severe combined immunodeficiency (SCID) is a rare, life-threatening immunologic genetic disorder resulting in profound T-cell deficiency with impaired T- and B-cell function. The phenotypic presentation of SCID can be variable, including additional B-cell and NK-cell deficiencies, but often exhibits strong genotype–phenotype correlations. One such phenotype, reticular dysgenesis (RD)—resulting from variants in *AK2*, includes additional physical and immunologic defects such as sensorineural hearing loss and neutropenia. Distinguishing RD from congenital neutropenia syndromes, bone marrow failure syndromes, and other immunodeficiencies based on phenotype may prove challenging, and confirmatory testing to identify a molecular cause is warranted.

Ras-related C3 botulinum toxin substrate (RAC) is a small GTP-binding protein within the Rho-GTPase family. There are three isoforms of RAC, with RAC2 being the dominant RAC protein expressed in hematopoietic cells. RAC2 is involved with various downstream cellular effector functions related to immune-mediated processes. Variants in *RAC2*, which result in immunodeficiency syndromes with wide phenotypic heterogeneity, have been increasingly recognized over the last decade. The total global incidence of *RAC2*-related immunodeficiency is unknown, and only 54 patients have been described (1).

In this report, we present a novel *RAC2* variant with an early RD-like phenotype and functional evidence of a constitutively active RAC2 protein. Our case is only the fifth patient (third identified variant) with a novel *RAC2* mutation to present with this unique phenotype (2, 3).

## Case description

A 1-week-old male was born full-term and small for gestational age (weight: 2,610 g—5th percentile) to non-consanguineous parents, with a birth history notable for maternal pre-eclampsia. Initial newborn hearing screen results were unavailable. The remainder of the immediate newborn period was unremarkable. Upon presentation to his primary care pediatrician at 6 days of life, he was found to be febrile (101.6°F) with associated scalp cellulitis located at the site of a previous scalp electrode used during delivery. He was hospitalized and started on broad-spectrum antimicrobials, including ampicillin, gentamicin, acyclovir, and vancomycin. Blood cultures and scalp wound cultures were positive for *Escherichia coli*, and following antibiotic sensitivity results, he was transitioned to single therapy with cefepime. Urine culture and serum human immunodeficiency virus (HIV) 1/2 RNA were negative. Additional scalp wound cultures, including fungal cultures and HSV-PCR surface cultures, were negative. Cell counts, protein, and glucose from lumbar puncture were unremarkable. A BioFire® meningitis/encephalitis panel was negative. Brain MRI was suspicious for osteomyelitis; thus, the cefepime dose was escalated for meningitis coverage. Repeat imaging 10 days later demonstrated soft tissue inflammatory changes without evidence of osteomyelitis.

The initial and repeat newborn screen results were reported as unsatisfactory/inconclusive. Newborn screen results in North Carolina have three possible outcomes based on total quantifiable T-cell receptor excision circles (TRECs)—normal, abnormal, and borderline. Unsatisfactory/inconclusive results generally occur due to sample collection errors or failure of internal controls. State-guided follow-up protocols vary depending on the result, with recollection routinely recommended in cases of unsatisfactory/inconclusive results. His white blood cell (WBC) count was profoundly reduced at 500 cells/ml, with a complete absence of neutrophils, an absolute lymphocyte count (ALC) of 400 cells/ml (80%), and an absolute monocyte count of 100 cells/ml (20%). Granulocyte colony-stimulation factor (G-CSF) was initially administered at 10 μg/kg/day without improvement in neutrophil counts and discontinued after 10 days. The immunoglobulin G level was normal at 413 mg/dl, with undetectable IgA and IgM. He developed thrombocytopenia (nadir 39,000 platelets/ml) without anemia and subsequently showed improvement in platelet counts.

Initial lymphocyte flow cytometry at day of life 16 showed a T-cell (CD3^+^) count of 320 cells/μl (78%), B-cell (CD19^+^) count of 13 cells/μl (3%), and NK-cell count of 68 cells/μl (17%). The naïve T-cell (CD3/CD45RA^+^) count was profoundly low at 26 cells/μl (15%), with an elevated CD3/CD45RO^+^ percentage of 83% (Table 1). A primary immunodeficiency next-generation sequencing panel was sent but was unable to be processed due to insufficient DNA. The patient was then transferred to our institution, given suspicion for SCID with T-/B-/NK- phenotype.

**Table 1:** Immunophenotype of the patient.

Subset	DOL 16 cells/μl (%)	DOL 26 cells/μl (%)	DOL 110 cells/μl (%)	Reference cells/μl (%)
ALC	**410**	**1,288**	**288**	3,400–9,000
CD3^+^	**320** **(78%)**	**1,034** **(80.3%)**	**201** (69.9%)	2,500–5,600 (51%–77%)
CD4^+^	**197** (48%)	**739** (57.4%)	**156** (54.0%)	1,600–4,000 (35%–64%)
CD8^+^	**121** **(29%)**	**286** (22.2%)	**44** (15.2%)	560–1,700 (12%–28%)
CD19^+^	**13** **(3%)**	**46** **(3.6%)**	**77** (26.9%)	300–3,000 (6%–41%)
NK cells	**68** (17%)	207 (16.1%)	**7** (**2.6%)**	170–1,100 (4%–18%)
CD3^+^/CD45RA^+^	**26** **(15%)**	**291** **(28.1%)**	**58** **(28.9%)**	1,200–3,700 (64%–95%)
CD3^+^/CD45RO^+^	150 **(83%)**	422 **(40.8%)**	**77** **(38.3%)**	90–1,200 (3%–31%)
CD4^+^/CD45RA^+^/CD62L^+^	NA	**146** **(19.7%)**	**59** **(38.0%)**	1,200–3,600 (61%–94%)

NA, not available.

Values outside the reference range (10th–90th percentile) are presented in bold (4).

**Figure 1:**
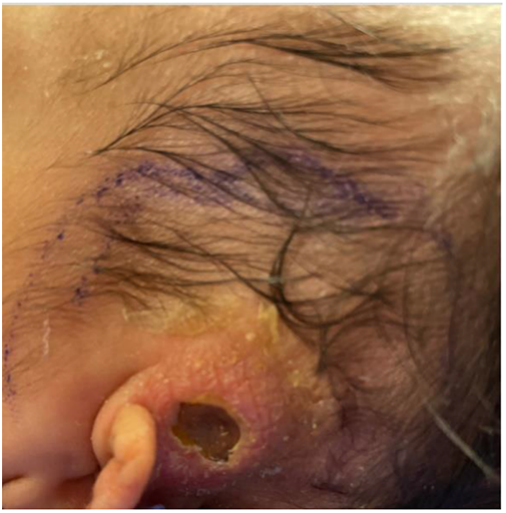
Scalp lesion. Evolving lesion located superior to the left ear of the patient notable for circumferential induration, absence of purulent material, and central necrosis/eschar formation.

Upon transfer, the patient was started on *Pneumocystis* and fungal prophylaxis with pentamidine and fluconazole, respectively. Mitogen proliferation to phytohemagglutinin (PHA) was normal. Maternal engraftment was not detected, and the red blood cell adenosine deaminase 1 (ADA) level was normal. Subsequent lymphocyte flow cytometry demonstrated an improvement in T- and NK-cell counts, with similarly low B-cell counts (Table 1). Multiple repeat newborn hearing screens were abnormal and thought to be associated with anatomical obstruction due to the scalp lesion (Figure 1). G-CSF was restarted and escalated to high-dose therapy (30 μg/kg/day) without notable improvement in neutrophil counts, so it was discontinued after 20 days. Bone marrow aspirate was obtained and showed no increased blasts, increased hematogones (29%), absence of maturing granulocytic elements (including neutrophil production), and active erythropoiesis without dysplasia. Bone marrow biopsy could not be obtained due to safety considerations related to the size of the patient.

## Diagnostic assessment

A repeat immunodeficiency gene panel collected via buccal swab was sent and revealed a novel heterozygous variant in *RAC2* [c.181C>A (p.Gln61Lys)] (Q61K). Based on its location within Switch region 2, this missense variant was expected to impact several RAC2 effector pathways. Despite early non-normal hearing screen results, his hearing screen normalized at 1 month of age.

Given the severity of the immune deficiency, early sepsis, and an identified underlying molecular defect, a haploidentical (maternal, variant negative) hematopoietic stem cell transplant was pursued at 4.5 months of age. Paternal donation was not a viable option due to a known underlying hematologic abnormality (phenotypically inconsistent with *RAC2* variants). The conditioning regimen consisted of alemtuzumab, fludarabine, and melphalan, along with post-transplant cyclophosphamide. The post-transplant course was complicated by the development of veno-occlusive disease (VOD) requiring defibrotide. One month following the transplant, he achieved >98% donor engraftment in CD3^+^, CD15^+^, and whole blood.

Immune reconstitution has been complicated (Table 2) by ongoing immunosuppressive therapies for managing graft-vs.-host disease (oral/topical calcineurin inhibitors, oral/topical corticosteroids, oral ruxolitinib, abatacept) and two episodes of Epstein–Barr virus (EBV) infection (treated with 4 weekly doses of rituximab). His most recent T-cell count is 735 cells/µl at 19 months post-transplant.

**Table 2:** Post-hematopoietic transplantation immune reconstitution.

Subset	3.5 months cells/μl (%)	10 months cells/μl (%)	19 months cells/μl (%)
ANC	570	1,000	1,400
ALC	**1,903**	**1,541**	**921**
CD3^+^	**944** (49.6%)	**1,415 (91.3%)**	**735 (80.2%)**
CD4^+^	**676** (35.5%)	**887 (57.6%)**	**453** (49.1%)
CD8^+^	**162** **(8.5%)**	**444** (28.8%)	**254** (27.6%)
CD19^+^	**523** (27.5%)	**0** ^a^	**45 (4.9%)** ^b^
NK-cells	403 **(21.2%)**	**103** (6.6%)	**119** (13.1%)
CD3^+^/CD45RA^+^	**365** **(38.7%)**	NA	NA
CD3^+^/CD45RO^+^	404 **(42.8%)**	NA	NA
CD4^+^/CD45RA^+^/CD62L^+^	**228** **(33.7%)**	NA	NA

NA, not available.

Lymphocyte values outside the reference range (10th–90th percentile) (4) are presented in bold.

^a^ 1 month post-rituximab.

^b^ 3 months post-rituximab.

He currently demonstrates mild developmental delays and is receiving speech and occupational therapy. A formal audiology assessment was completed at around 1 year of age and was normal. He receives nutrition via a gastrostomy tube. Growth has been stable but markedly below normal, with weight and height at the <1st and 2nd percentiles, respectively.

## Discussion

Ras-related C3 botulinum toxin substrate (RAC) is a small GTP-binding protein within the Rho-GTPase family (5). GTPases impact various downstream cellular functions by switching between active and inactive states induced through GTP/GDP binding (6). Within the RAC family, three homologous isoforms have been identified: RAC1, RAC2, and RAC3 (5, 7). RAC1 is ubiquitously expressed, and RAC3 is neuronally expressed, while RAC2 is the dominant RAC protein in hematopoietic cells. As such, it plays a fundamental role in immune-mediated cellular effector functions, including cell migration, cytoskeletal reorganization, and neutrophil superoxide production, although the specifics of these mechanisms are still being elucidated (8, 9). *RAC2* variants demonstrate phenotypic heterogeneity across dominant, constitutively activating, dominant activating, dominant negative, and autosomal recessive subtypes (2, 6, 10, 11). Thus, the clinical spectrum of disease can be quite variable, as described by Donkó et al. (1), including later-onset combined immunodeficiency (CVID) with normal or depressed neutrophil counts (10, 12–16), leukocyte-adhesion deficiency-like disease with normal or elevated neutrophil counts (11, 17), and severe combined immunodeficiency RD-like disease with neutropenia but lacking sensorineural hearing loss (2, 3).

RD is caused by variants in *AK2*, which encodes adenylate kinase 2, a vital enzyme in the production of cellular energy through phosphorus transfer to produce adenosine triphosphate (ATP) (18). An obligatory, non-hematopoietic, clinical feature of RD is sensorineural hearing loss. However, the pathophysiologic mechanisms, either developmental or functional, related to hearing loss are not well understood. Importantly, despite the immunologic similarities of some *RAC2* activating mutations to RD, sensorineural hearing loss does not appear to be a phenotypic feature (2, 3). Thus, hearing evaluation should be considered an early priority to assist in differentiating between variants presenting as RD or RD-like.

**Figure 2:**
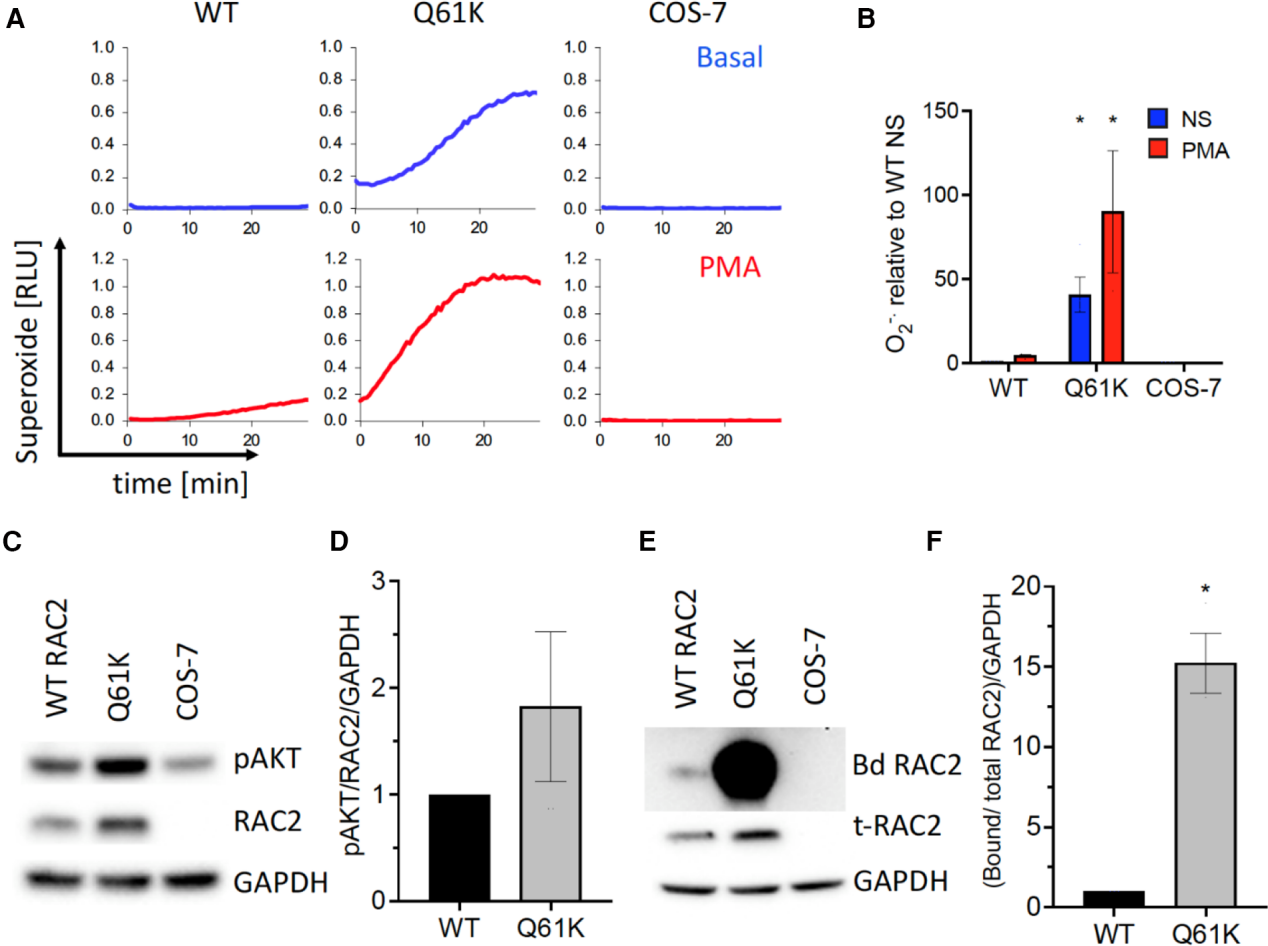
RAC2-based functional assays of superoxide production, AKT phosphorylation, and PAK1 binding. (**A**) Extracellular reactive oxygen species production for COS-7 cells transfected with NADPH oxidase components gp91^phox^, p67^phox^, p47^phox^, and wild-type (WT), Q61K variant, or untransfected (COS-7) over 30 min under basal and PMA-stimulated conditions. (**B**) Summary bar graph of integrated kinetics of basal and PMA-stimulated superoxide production normalized to WT RAC2 non-stimulated, expressed as mean ± SEM from three independent experiments. (**C**) Q61K variant showing increased AKT phosphorylation consistent with enhanced constitutive RAC2 activation; representative Western blot. (**D**) Summary of AKT phosphorylation from three independent experiments analyzed by densitometry, normalized to WT and GAPDH levels, expressed as average ± SEM. (**E**) Q61K variant showing a significant increase in Western blot of PAK1-bound RAC2 (Bd RAC2) from wild-type or Q61K-transfected or non-transfected (COS-7) cells. (**F**) Quantification of the PAK1-bound RAC2 Q61K variant showing significantly higher bound RAC2 than wild-type RAC2, consistent with increased constitutive activity.

Identification of a novel *RAC2* variant in this patient prompted research-based testing to assess RAC2 activity and the impact on cellular effects, including superoxide production and membrane ruffling, which have previously been described (1). Using a variant expression construct and heterologous expression system, the Q61K variant function was compared to wild-type RAC2. The methodological details are outlined by Donkó et al. (1). The Q16K variant supported elevated superoxide production by NOX2 in reconstituted cells under both basal (40-fold higher than wild-type) and PMA-stimulated (5-fold higher than wild-type) conditions (Figures 2A,B). In addition, PAK1 binding was assessed as a measure of downstream activity (GTP-bound RAC2) and demonstrated 15-fold higher binding (Figures 2C–F). Together, these findings were consistent with other dominant, constitutively activated *RAC2* mutations.

On initial presentation, our patient's clinical phenotype matched that of patients reported by Stern et al. and Lagresle-Peyrou et al. who had agranulocytosis with a T-/B-/NK- SCID phenotype associated with *RAC2* variants: [c.182 A>G (p.Gln61Arg)] (Q61R) and [c.34 G>A, (p.Gly12Arg)] (G12R) (2, 3). However, subsequently, T-cell counts increased to above 1,000 cells/μl, and mitogen proliferative response to PHA was normal. Thus, this patient did not meet the Primary Immunodeficiency Treatment Consortium's (PIDTC) criteria for typical or leaky/atypical SCID (19). In addition to leukopenia with normal red cell and platelet counts noted shortly after birth, at the time of presentation to the outside tertiary care center, he was diagnosed with pancytopenia, consistent with sepsis. Prior to the initiation of his transplant preparation, leukopenia persisted with mild anemia and normal platelet counts. Altogether, these features raised concern for an alternative explanation for the phenotype of the patient, including syndromes associated with congenital neutropenia, bone marrow failure, or other causes of immune dysregulation. In retrospect, we speculate that the change in immunophenotype (improvement in lymphocyte counts) was reflective of high-dose G-CSF driving additional T-cell and NK-cell production at the time of flow cytometry evaluation. Randomized, placebo-controlled trials have demonstrated increased lymphocyte production in response to G-CSF among adult patients with SARS-CoV2 infection (ALC <800 cells/ml) as well as patients with HIV1 receiving antiretroviral therapy (ALC <350 cells/ml) (20, 21). In the HIV1 trial, increased CD4^+^ and CD8^+^ production was primarily of a memory (CD45RO^+^) phenotype (20), which may have been representative of an oligoclonal expansion, although clonality testing was not explored. Interestingly, this patient demonstrated an increase in both the naïve (CD45RA^+^) and memory T-cell compartment, although a higher proportion of memory T-cells overall was maintained. However, the total naïve T-cell percentage (28.1%) remained low for age (ref: 61%–94%) (4). Upon discontinuing G-CSF, T-cell counts (CD3^+^) decreased to 201 cells/μl. Despite *RAC2* being classified as a SCID-related gene, the phenotype of this patient remained inconsistent with PIDTC criteria for typical and atypical/leaky SCID based on the following: CD3^+^ count, naïve T cells >20% of total CD4^+^ count, normal proliferative studies, no evidence of maternal engraftment, absence of T-cell clonality studies, and inconclusive SCID screen results (inhibiting TREC quantification) (19).

The SCID newborn screen results of the patient are particularly noteworthy given the challenge of interpreting unsatisfactory and inconclusive results. While there are clear guidelines for follow-up evaluation (or lack thereof) in cases of normal, borderline, and abnormal results, there is much more uncertainty in cases with unsatisfactory and inconclusive results. Unsatisfactory results occur when samples are unacceptable for processing at the state lab (e.g., uneven blood spotting on Guthrie cards), generally prompting recommendations to send a repeat sample. Inconclusive results are generated when there is a disruption in the internal RNase controls and may represent a technical or sample processing error. However, inconclusive results may be caused by severe cytopenia, reflecting an inability of the RNase primer to bind due to depressed or absent sample DNA. Identifying the basis for inconclusive results can be difficult, often requiring discussion with the state laboratory in cases of repetitive inconclusive reporting. Complete blood counts with differentials may represent an early and simple screening option to assess leukocyte counts and identify aberrations requiring evaluation.

Our case highlights the diagnostic challenges associated with genetic variants of inborn errors of immunity. Although our patient did not meet the recent 2022 PIDTC criteria for SCID, the immunologic impairment warranted HSCT. Constitutively activating *RAC2* mutations have been implicated in an RD-like SCID phenotype, and the initial presentation of our patient was consistent with this clinical picture. However, subsequent testing with improved (but still low) lymphocyte counts suggests that the patient had a combined immunodeficiency phenotype with a neutrophil defect. The details of our case reinforce the importance of confirmatory gene sequencing, especially among phenotypically variable immune defects, and the importance of functional assays to investigate aberrant pathways confirming variant pathogenicity. Furthermore, continuing reports on patients with *RAC2* variants will be paramount in characterizing disease phenotype and outcomes following definitive therapy.

## Data Availability

Anonymized data may be available upon request. Functional assays completed by co-authors at the NIH. Genetic results are provided through commercially available next-generation sequencing panels as part of the standard of care for such patients. Requests to access the datasets should be directed to talal.mousallem@duke.edu, tleto@niaid.nih.gov, or agnes.donko@nih.gov.
